# SOHLH2-RAD54L axis induces radioresistance by promoting homologous recombination repair in non-small cell lung cancer

**DOI:** 10.1038/s41420-025-02924-9

**Published:** 2026-01-14

**Authors:** Jia-Xue Yang, Wei-Hua Zhang, Jin-Ju Lei, Chun Cheng, Meng Yu, Ping Zhang, Yi Sang

**Affiliations:** 1 Jiangxi Key Laboratory of Oncology, Department of Center Laboratory, The Third Affiliated Hospital of Nanchang University, Nanchang, China; 2 Department of Oncology, Yangxin People’s Hospital, Yangxin, Hubei China; 3 Integrated Traditional Chinese and Western Medicine Oncology Department, Jiangxi Provincial People’s Hospital, Nanchang, Jiangxi China; 4 Cancer Center, Renmin Hospital of Wuhan University, Wuhan, Hubei China

**Keywords:** Oncogenesis, DNA damage and repair

## Abstract

Radiation resistance is the major cause of non-small cell lung cancer (NSCLC) treatment failure. Homologous recombination (HR), which mediates the repair of DNA double-strand breaks (DSB), is crucial for maintaining genomic integrity and enhancing survival in response to radiotherapy in NSCLC. However, the mechanisms of HR repair in radiation resistance remains unclear. In this study, we investigated the functional role of the transcription factor *Spermatogenesis and oogenesis basic helix-loop-helix transcription factor 2* (SOHLH2) in NSCLC HR repair and radioresistance. Our research unveiled that the expression levels of SOHLH2 increased in NSCLC compared with adjacent non-tumor tissues. Elevated SOHLH2 expression promotes NSCLC cell proliferation and radiation resistance, while knocking down SOHLH2 has the opposite effect. Mechanistically, SOHLH2 transcriptionally activated the expression of RAD54L, thereby promoting HR repair and the survival of cancer cells in response to radiation. Notably, RAD54L overexpression was able to rescue the suppression of NSCLC HR repair and radioresistance induced by SOHLH2 knockdown. Therefore, SOHLH2-RAD54L axis may serve as a potential therapeutic target for overcoming radioresistance in NSCLC.

## Introduction

Lung cancer is one of the most common malignancies and remains a leading cause of cancer-related mortality worldwide [1–3]. The incidence and mortality rates of lung cancer continue to rise, posing a significant global public health challenge [4, 5]. Non-small cell lung cancer (NSCLC) is the predominant subtype, accounting for approximately 85% of all lung cancer cases [6]. Based on histopathological features, NSCLC can be classified into three major subtypes: adenocarcinoma, squamous cell carcinoma, and large cell carcinoma [7]. Substantial progress has been made in the treatment of NSCLC through surgical resection, chemotherapy, targeted therapies, and immunotherapy [8, 9]. In particular, radiotherapy has yielded favorable clinical outcomes for a subset of patients in recent years [10]. However, the development of radioresistance in tumor cells remains a major obstacle, compromising therapeutic efficacy and prognosis in many NSCLC patients [11]. Therefore, elucidating the mechanisms underlying NSCLC proliferation and radioresistance is of critical importance for improving patient outcomes.

Spermatogenesis and oogenesis basic helix-loop-helix transcription factor *2* (SOHLH2), located on chromosome 13, is a member of the basic helix-loop-helix (bHLH) transcription factor family [12–14], and is highly expressed in germ cells [15]. Recent studies have demonstrated that SOHLH2 plays an essential role in tumorigenesis and cancer progression [16, 17]. However, the specific role of SOHLH2 in NSCLC remains largely unexplored.

RAD54L, a member of the Swi2/Snf2 ATP-dependent chromatin remodeling family, has been identified as a key factor in the homologous recombination repair (HRR) pathway [18, 19]. Increasing evidence indicates that RAD54L plays a critical role in HR, which is a major mechanism for the repair of DNA double-strand breaks [20, 21]. As radioresistance is closely associated with the DNA damage repair capacity of tumor cells, the HR pathway has attracted growing attention in this context [22]. RAD54L, as a vital component of HRR, has been implicated in the regulation of radioresistance and has been shown to play a pivotal role in this process [23, 24].

This study was designed to elucidate how SOHLH2 regulates RAD54L expression to promote proliferation and radioresistance in NSCLC cells. In vitro experiments, gene expression analyses, and molecular biology techniques were employed to systematically explore the regulatory role of SOHLH2 in relation to RAD54L. It is anticipated that these findings will provide novel insights into the SOHLH2–RAD54L axis in NSCLC, potentially offering new therapeutic avenues to overcome radioresistance and improve patient prognosis and quality of life.

## Results

### Result 1. SOHLH2 is highly expressed in NSCLC

**Fig. 1:**
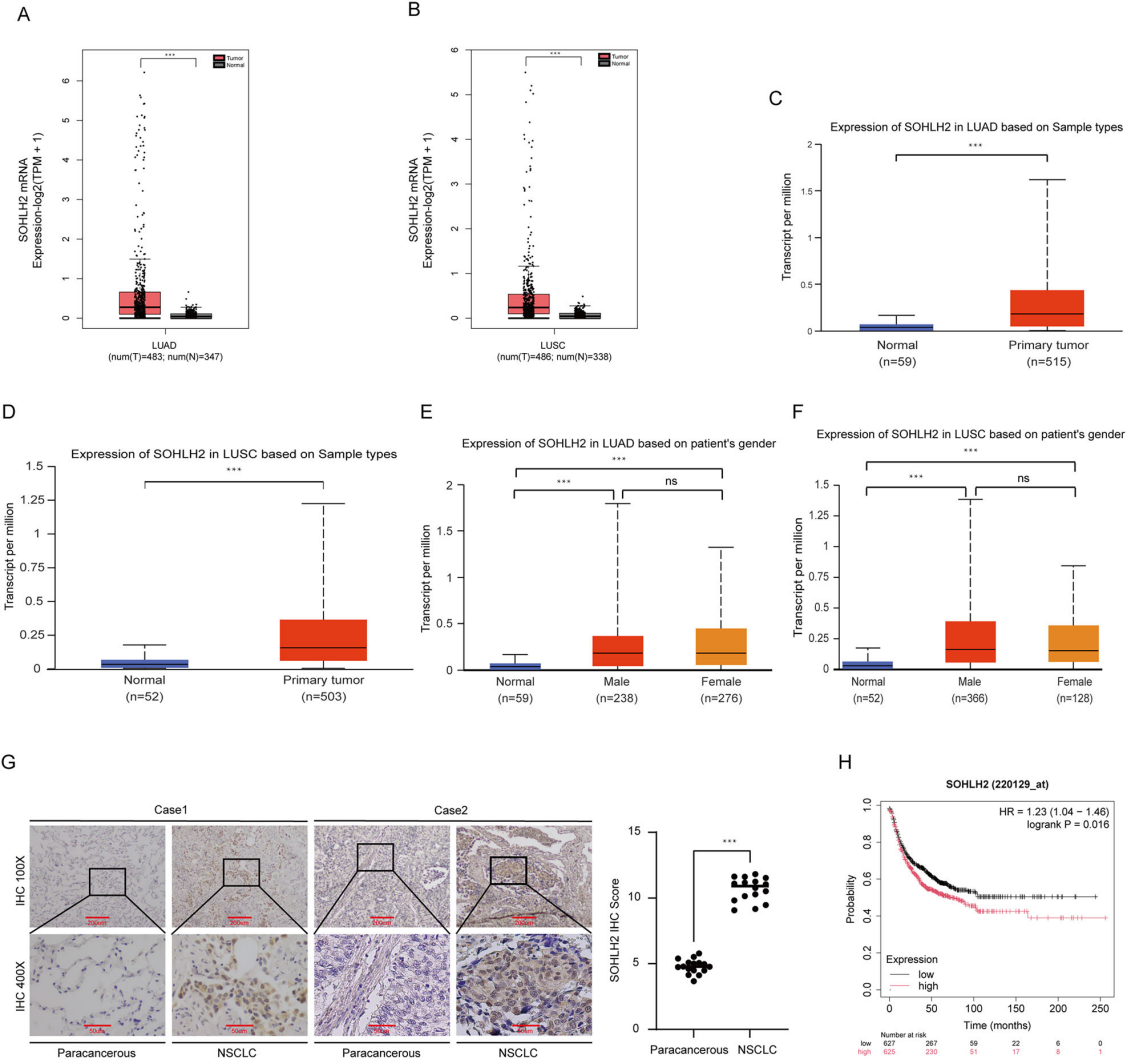
SOHLH2 is highly expressed in NSCLC and predicts poor outcomes. **A**, **B** GEPIA database analysis indicates that SOHLH2 mRNA expression is significantly elevated in non-small cell lung cancer (NSCLC) tissues compared to normal lung tissues. **C**, **D** UALCAN database analysis based on TCGA datasets confirms the upregulation of SOHLH2 mRNA in NSCLC. **E**, **F** Gender-based analysis using TCGA cohorts shows no significant difference in SOHLH2 mRNA expression between male and female patients in both LUAD and LUSC subtypes. **G** Representative immunohistochemical (IHC) staining images (scale bars: 200 µm and 50 µm) and corresponding IHC scores for SOHLH2 expression in 16 paired NSCLC and adjacent non-tumor tissues. SOHLH2 protein levels were significantly higher in tumor tissues compared to adjacent tissues. **H** Kaplan–Meier survival analysis reveals that patients with high SOHLH2 expression exhibit significantly shorter first progression (FP) survival than those with low SOHLH2 expression (*P* < 0.05). ***P < 0.001.

The expression profile of SOHLH2 was analyzed using two bioinformatics platforms: GEPIA (Gene Expression Profiling Interactive Analysis) and UALCAN. According to the GEPIA database, SOHLH2 mRNA expression was significantly elevated in lung adenocarcinoma (LUAD, log2FC = 1, *p* < 0.05) and lung squamous cell carcinoma (LUSC, log2FC = 1, *p* < 0.05) compared with normal lung tissues (Fig. 1A, B). Consistently, data retrieved from the UALCAN database (TCGA dataset) confirmed higher SOHLH2 expression in both LUAD (*p* < 0.001; Fig. 1C) and LUSC tissues (*p* < 0.001; Fig. 1D) relative to their respective normal controls. In LUAD, there was no statistically significant difference in SOHLH2 expression between male (*n* = 238) and female patients (*n* = 276) (*p* = 0.482; Fig. 1E). A similar trend was observed in LUSC (male *n* = 366 vs female *n* = 128, *p* = 0.985; Fig. 1F). These findings suggest that SOHLH2 may function as a potential oncogenic driver in NSCLC (*p* > 0.05).

Next, we assessed SOHLH2 protein expression in NSCLC tissues (*n* = 16) and corresponding adjacent non-tumor tissues (*n* = 16) using immunohistochemistry (IHC). Slides were scanned and quantitatively analyzed using ImageJ 2.0 software (NIH, USA). Two independent pathologists, blinded to the experimental grouping, performed scoring. Staining intensity (SI) was scored on a scale of 0–3: 0 (negative), 1 (weak), 2 (moderate), and 3 (strong), while the percentage of positively stained cells (PPC) was graded as follows: 0 (0%), 1 (1–10%), 2 (11–50%), 3 (51–80%), and 4 (81–100%). A composite score was calculated (SI × PPC, ranging from 0 to 12), with the expression levels defined as follows: negative (–, score 0–1), weak (+, score 2–4), moderate (++, score 5–8), and strong (+++, score 9–12). Scoring criteria were based on the Guidelines for Interpretation of Immunohistochemical Results (2020 Edition). Inter-rater reliability analysis using the intraclass correlation coefficient (ICC) showed excellent agreement between the two pathologists for both SI (ICC = 0.85) and PPC (ICC = 0.82), with an overall Cohen’s kappa value of 0.83 (95% CI: 0.78–0.88), indicating strong consistency. Results demonstrated a significant increase in SOHLH2 protein expression in NSCLC tissues compared to adjacent non-tumor tissues (Fig. 1G). Furthermore, Kaplan–Meier survival analysis (http://kmplot.com/analysis/) revealed that high SOHLH2 expression was significantly associated with shorter first progression survival (FP) in lung cancer patients (Fig. 1H). Collectively, these findings indicate that SOHLH2 is upregulated in NSCLC irrespective of patient gender, and its high expression is correlated with poor prognosis.

### Result 2. SOHLH2 promotes proliferation and radioresistance in non-small cell lung cancer cells

**Fig. 2:**
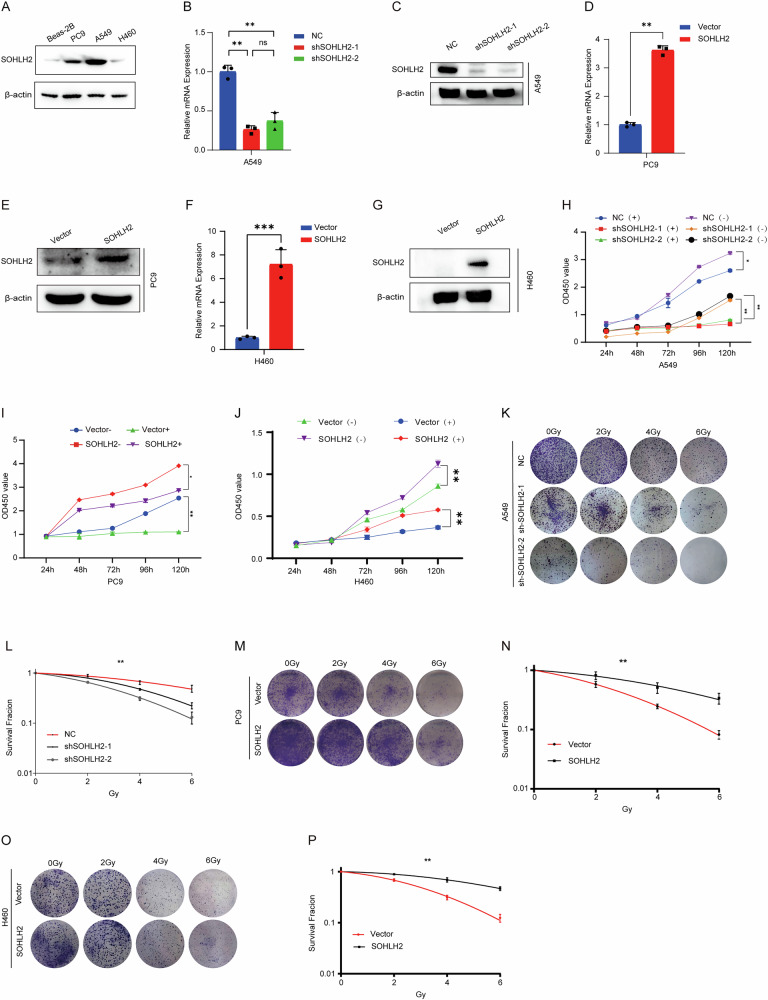
SOHLH2 promotes proliferation and radioresistance in non-small cell lung cancer cells. **A** Western blot analysis of SOHLH2 protein expression in NSCLC cell lines (A549, PC9, and H460) and Beas-2B cells. **B** Quantitative PCR (qPCR) analysis showing a significant decrease in SOHLH2 mRNA levels in A549 cells following knockdown. **C** Western blot analysis showing a marked reduction in SOHLH2 protein levels in A549 cells after knockdown. **D** qPCR analysis showing significantly increased SOHLH2 mRNA levels in PC9 cells after overexpression. **E** Western blot analysis confirming elevated SOHLH2 protein expression in PC9 cells following overexpression. **F** qPCR analysis showing significantly increased SOHLH2 mRNA levels in H460 cells after overexpression. **G** Western blot analysis confirming elevated SOHLH2 protein expression in H460 cells following overexpression. **H**–**J** Cell viability assay results of A549、PC9 and H460 cells after exposure to 0 or 4 Gy ionizing radiation (IR). Data are presented as mean ± SD, analyzed using Student’s unpaired two-sided *t*-test. **K**–**P** Clonogenic survival assays of A549, PC9 and H460 cells after exposure to 0, 2, 4, and 6 Gy IR. Representative images are shown in (**K**), (**M**) and (**O**). The above data are presented as the mean ± SD. of three independent experiments and analyzed by two-way ANOVA. *P < 0.05, **P < 0.01.

To further investigate the functional role of SOHLH2 in non-small cell lung cancer (NSCLC), We compared the expression levels of SOHLH2 in the non-small cell lung cancer cell lines of A549, PC9, and H460 and in the normal lung epithelial cell line of BEAS-2B.SOHLH2 was found to be highly expressed in A549, PC9 and H460 cells (Fig. 2A) ; thus, these three cell lines were selected for subsequent experiments. We established A549 cells with stable knockdown of SOHLH2 (Fig. 2B, C), PC9 and H460 cells with stable overexpression of SOHLH2 (Fig. 2D–G). Cell Counting Kit-8 (CCK-8) assays and colony formation assays were then performed to evaluate the impact of SOHLH2 on tumor cell proliferation and radioresistance in NSCLC. Following X-ray irradiation, A549 cells with SOHLH2 knockdown exhibited significantly reduced proliferative capacity and survival rates compared to control cells. Conversely, PC9 and H460 cells with SOHLH2 overexpression showed increased proliferation and survival following irradiation (Fig. 2H–P). These findings suggest that SOHLH2 enhances both the proliferative ability and radioresistance of NSCLC cells.

### Result 3. SOHLH2 may mediate radioresistance in NSCLC by regulating homologous recombination repair of DNA damage

**Fig. 3:**
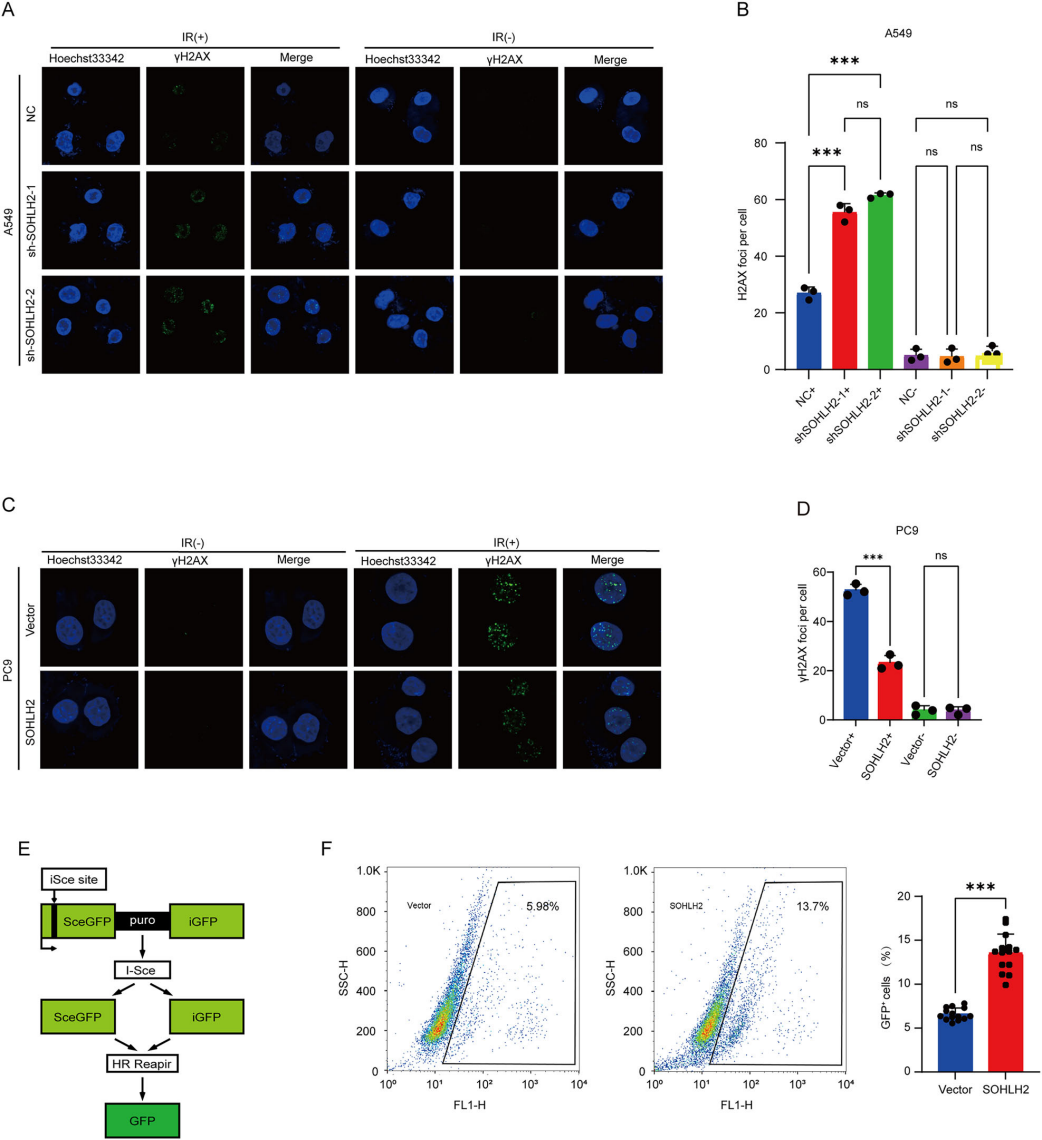
SOHLH2 promotes radioresistance in NSCLC by enhancing homologous recombination repair of DNA damage. **A**, **C** Immunofluorescence staining of γ-H2AX was performed in A549 and PC9 cells after exposure to 0 Gy or 6 Gy of X-ray irradiation. Knockdown of SOHLH2 in A549 cells increased γ-H2AX foci formation, whereas SOHLH2 overexpression in PC9 cells decreased γ-H2AX foci. Nuclei were counterstained with Hoechst 33342 (blue), and γ-H2AX foci were visualized in green. Scale bar = 20 µm. **B**, **D** Quantification of γ-H2AX foci per nucleus. Data are presented as mean ± SD and analyzed using Student’s unpaired two-tailed t test. **P < 0.01, ***P < 0.001. **E** Schematic representation of the DR-GFP reporter construct used to assess homologous recombination activity. **F** Flow cytometry analysis of GFP-positive cells in HeLa-DR-GFP cells 48 h after co-transfection with I-SceI and SOHLH2 expression plasmids by electroporation. Overexpression of SOHLH2 significantly increased HR efficiency. The above data are presented as the mean ± SD. of three independent experiments. **P < 0.01, ***P < 0.001.

Ionizing radiation (IR) primarily induces DNA damage in the form of DNA double-strand breaks (DSBs) [25, 26]. In response to DSBs, histone H2AX is phosphorylated to form γ-H2AX, which rapidly accumulates at DNA break sites [27]. This modification recruits various DNA damage response (DDR) factors to form repair foci and initiate the repair process [27]. γH2AX is a sensitive marker for DSBs [28]. To investigate the role of SOHLH2 in DNA damage repair, we performed immunofluorescence assays and observed that SOHLH2 knockdown in A549 cells significantly increased the number of γ-H2AX foci following IR exposure (Fig. 3A, B), whereas SOHLH2 overexpression in PC9 cells markedly reduced γ-H2AX foci (Fig. 3C, D).We further employed a DR-GFP reporter assay to assess homologous recombination (HR) efficiency. Results showed a higher proportion of GFP-positive cells in the SOHLH2-overexpressing group compared to the vector control group (Fig. 3E, F), indicating enhanced HR activity. Collectively, these findings suggest that SOHLH2 may facilitate DNA damage repair through homologous recombination and thereby contribute to radioresistance in non-small cell lung cancer.

### Result 4. SOHLH2 Directly increased the Expression of RAD54L

**Fig. 4:**
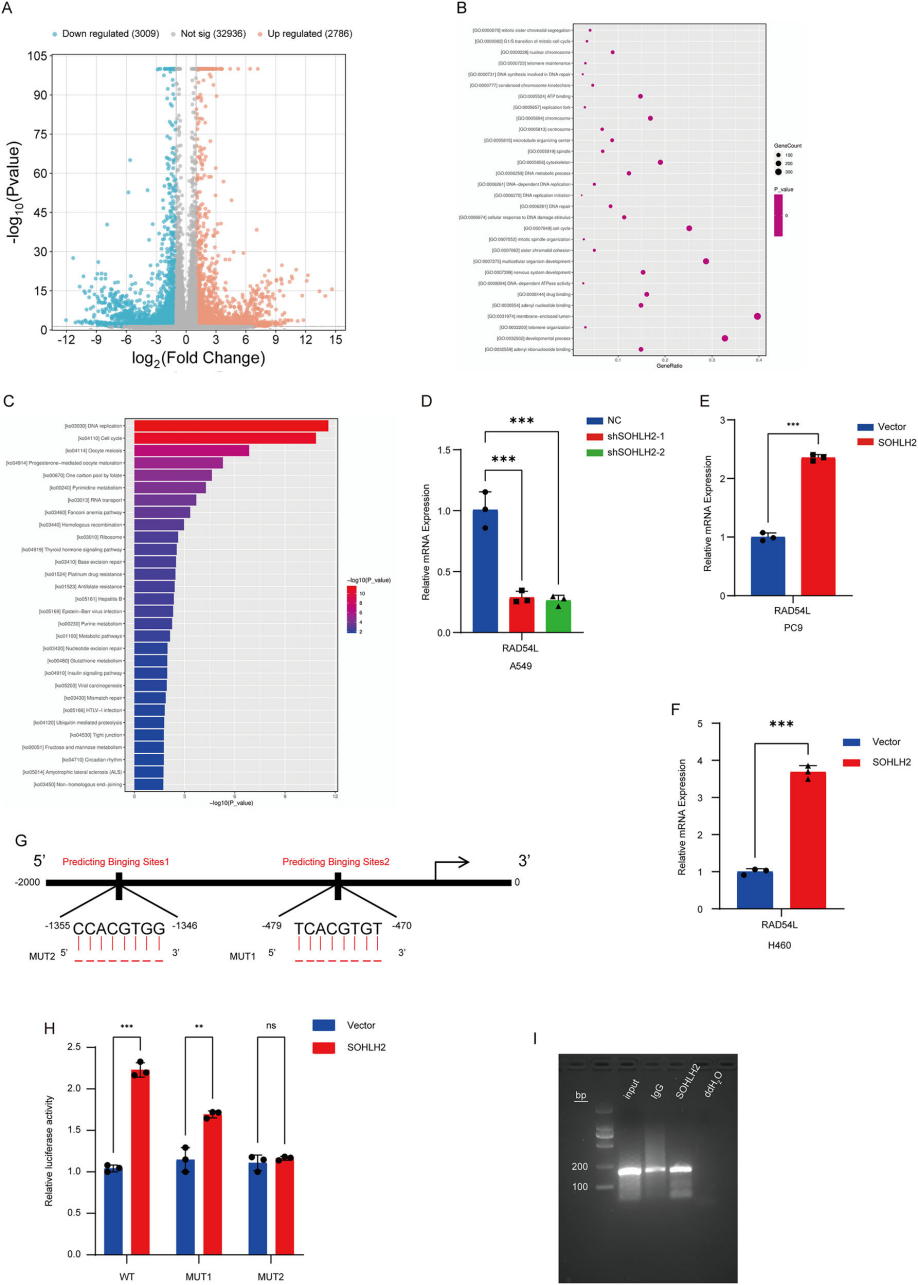
SOHLH2 directly regulates the expression of RAD54L. **A** Volcano plot of differentially expressed genes (DEGs): Orange dots represent significantly upregulated genes, green dots represent significantly downregulated genes, and gray dots represent genes with no significant change. **B** GO enrichment analysis: The X-axis shows the enrichment factor, and the Y-axis shows the associated pathways/biological processes. Dot size indicates the number of genes enriched in each term, and larger dots denote higher statistical significance. **C** KEGG enrichment analysis: The *X*-axis shows the enrichment factor, and the *Y*-axis shows the enriched pathways. Bar length reflects the number of genes involved in each pathway, and the color indicates statistical significance. **D** RT-qPCR results showing that RAD54L expression was decreased upon SOHLH2 knockdown in A549 cells. **E**, **F** RT-qPCR analysis showed that RAD54L expression was increased upon SOHLH2 overexpression in PC9 and H460 cells. **G** Bioinformatic prediction of SOHLH2 binding sites in the RAD54L promoter region. Two mutants were constructed: MUT1 with deletion at −478 to −471 and MUT2 with deletion at −1355 to −1347. **H** Dual-luciferase reporter assay confirming RAD54L as a direct target of SOHLH2. **I** Gel electrophoresis assay showing that SOHLH2 is enriched at the -1362 to -1373 region of the RAD54L promoter in A549 cells. The above data are presented as the mean ± SD. of three independent experiments. **P < 0.01, ***P < 0.001.

To elucidate the molecular mechanism by which SOHLH2 mediates radioresistance in NSCLC, RNA sequencing (RNA-seq) was performed following SOHLH2 knockdown, conducted by Shanghai Yuanxin Biopharmaceutical Technology Co., Ltd. Differentially expressed genes (DEGs) were identified based on the criteria of |log₂(Fold Change)| ≥ 0.5 and *p* < 0.05, revealing 2,787 upregulated and 3,009 downregulated genes (Fig. 4A). Gene Ontology (GO) enrichment analysis of the downregulated DEGs demonstrated significant enrichment in DNA damage repair pathways (Fig. 4B), while KEGG pathway enrichment analysis showed that a subset of downregulated genes was significantly enriched in homologous recombination (HR) pathways (Fig. 4C). Among these genes, we focused on RAD54L, a key factor involved in homologous recombination repair.

Subsequently, RT-qPCR validation revealed that RAD54L expression was decreased upon SOHLH2 knockdown in A549 cells (Fig. 4D), while its expression was elevated following SOHLH2 overexpression in PC9 and H460 cells (Fig. 4E, F). These results indicate that SOHLH2 increased RAD54L expression.

To further determine whether RAD54L is a direct transcriptional target of SOHLH2, we predicted SOHLH2 binding sites in the promoter region of RAD54L (Fig. 4G). Luciferase reporter plasmids containing wild-type (RAD54L-WT) or mutant (RAD54L-MUT1 and RAD54L-MUT2) 3’UTR sequences were constructed. Dual-luciferase reporter assays demonstrated that SOHLH2 significantly enhanced luciferase activity in both RAD54L-WT and RAD54L-MUT1 constructs, but not in RAD54L-MUT2 (Fig. 4H), suggesting that the MUT2 region contains the SOHLH2 binding site. To confirm this interaction, chromatin immunoprecipitation (ChIP) assays were performed using SOHLH2 antibody, with IgG as a negative control and input as a positive control. Gel electrophoresis results showed that SOHLH2 was significantly enriched at the predicted binding site 2 of the RAD54L promoter in A549 cells (Fig. 4I). Collectively, these results demonstrate that SOHLH2 directly regulates RAD54L expression by binding to its promoter region.

### Result 5. Overexpression of RAD54L reverses the inhibitory effects of SOHLH2 knockdown on NSCLC cell proliferation and radioresistance

**Fig. 5:**
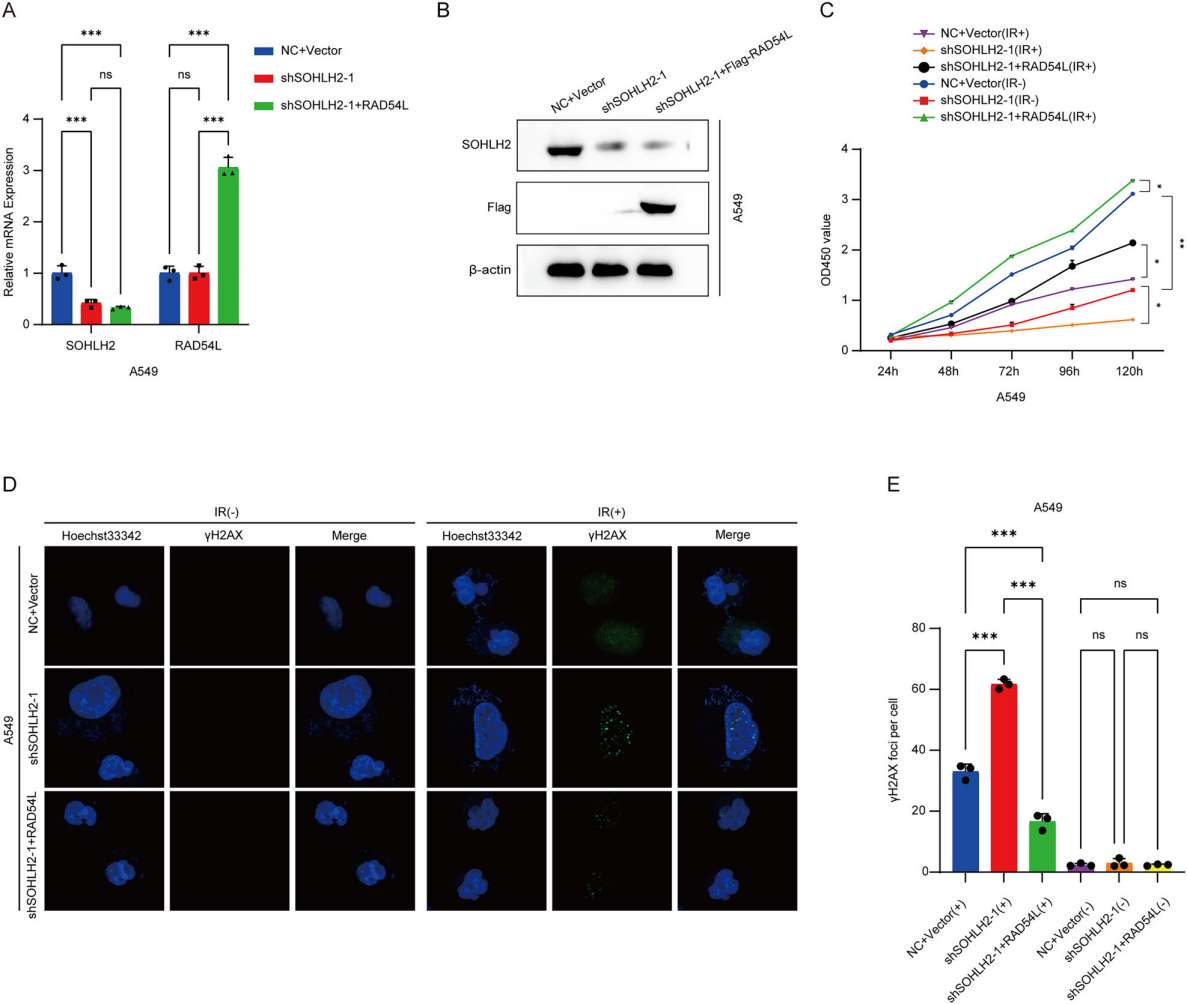
Overexpression of RAD54L reverses the inhibitory effects of SOHLH2 knockdown on NSCLC cell proliferation and radioresistance, and promotes DNA damage in NSCLC cells after radiotherapy. **A** The mRNA expression levels of SOHLH2 knockdown in A549 cells and RAD54L overexpression in SOHLH2-knockdown A549 cells were detected by qPCR. **B** The protein expression levels of SOHLH2 knockdown in A549 cells and RAD54L overexpression in SOHLH2-knockdown A549 cells were detected by Western blotting. **C** CCK-8 assay was performed to evaluate the reversal effect of RAD54L overexpression on the inhibition of NSCLC cell proliferation and radioresistance caused by SOHLH2 knockdown. **D**, **E** Immunofluorescence assay was conducted to assess the promoting effect of RAD54L overexpression on DNA damage in NSCLC cells after radiotherapy following SOHLH2 knockdown. The above data are presented as the mean ± SD. of three independent experiments. *P < 0.05, **P < 0.01, ***P < 0.001.

To elucidate the functional mechanism of SOHLH2 in NSCLC, we established a stable A549 cell line overexpressing RAD54L in the context of SOHLH2 knockdown (Fig. 5A, B). Following X-ray irradiation, A549 cells with SOHLH2 knockdown exhibited significantly reduced proliferation and survival rates compared to the control group. However, these effects were markedly attenuated by RAD54L overexpression (Fig. 5C). These findings indicate that RAD54L overexpression can rescue the inhibitory effects of SOHLH2 knockdown on NSCLC cell proliferation and radioresistance.

### Result 6. Overexpression of RAD54L attenuates the SOHLH2 knockdown-induced increase in radiation-induced DNA damage in NSCLC cells

To investigate whether the enhanced DNA damage observed after radiotherapy in SOHLH2-deficient NSCLC cells is mediated by RAD54L, we performed immunofluorescence staining. In A549 cells, SOHLH2 knockdown significantly increased the number of γ-H2AX foci following ionizing radiation (IR), indicating elevated DNA damage. However, this increase was significantly reduced upon RAD54L overexpression (Fig. 5D, E). These results suggest that RAD54L overexpression mitigates the radiation-induced DNA damage promoted by SOHLH2 knockdown in NSCLC cells.

## Discussion

Non-small cell lung cancer (NSCLC) is the most common type of lung cancer, with its incidence and mortality rates continuously rising, making it a leading cause of cancer-related deaths [29, 30]. Despite ongoing advances in medical technology and improvements in treatment modalities, early diagnosis and therapeutic efficacy for NSCLC remain challenging [31, 32]. Currently, combined application of surgery, radiotherapy, chemotherapy, and targeted therapies can improve treatment outcomes to some extent [33]. However, resistance to radiotherapy remains a major obstacle affecting the efficacy of NSCLC treatment [34]. Understanding the underlying biological mechanisms of this resistance is critical for improving patient prognosis and developing novel therapeutic strategies.

SOHLH2 is a unique transcription factor that plays a key regulatory role in the progression of various malignancies and has been identified as a promising target in cancer therapy. Studies have demonstrated that SOHLH2 significantly influences the occurrence and progression of breast cancer [17]. Additionally, SOHLH2 is involved in the proliferation, migration, and invasion of ovarian cancer cells [35–37]. For instance, SOHLH2 was found to inhibit ovarian cancer cell proliferation by regulating the expression of cell cycle-related proteins [37]. Another study revealed that SOHLH2 suppresses renal cancer cell proliferation through modulation of DNMT3a/Klotho expression [38]. These findings suggest that SOHLH2 may function as a tumor suppressor by regulating the proliferation and differentiation of malignant cells.

In contrast, our study indicates a different role of SOHLH2 in NSCLC. Analysis of public databases and immunohistochemical experiments revealed a significant upregulation of SOHLH2 expression in NSCLC tissues. This finding contradicts observations in other tumor types and suggests that SOHLH2 may promote tumor progression in NSCLC. To validate this, CCK-8 and colony formation assays were performed. The results demonstrated that the proliferative capacity and colony-forming ability of SOHLH2 knockdown cells were significantly reduced compared to the vector control group, regardless of X-ray irradiation. Subsequently, PC9 (KRAS wild-type) and H460 (KRAS mutant) stable cell lines overexpressing SOHLH2 were established. In contrast to the knockdown results, SOHLH2 overexpression significantly enhanced cell proliferation and radioresistance. Notably, these pro-proliferative and radioresistant effects of SOHLH2 were consistently observed in both KRAS wild-type and mutant contexts, indicating its role is independent of KRAS status. These results collectively indicate that SOHLH2 substantially promotes the proliferative process in NSCLC cells.

Radiotherapy is a critical treatment modality for NSCLC; however, tumor cell radioresistance limits its effectiveness [39]. Radioresistance remains a major challenge in NSCLC treatment, markedly diminishing therapeutic outcomes [40, 41]. Therefore, elucidating the mechanisms underlying NSCLC radioresistance has become a focus of current cancer research. To further investigate the effect of SOHLH2 on NSCLC radioresistance, CCK-8 and clonogenic assays were conducted. The results showed that SOHLH2 knockdown inhibited NSCLC radioresistance, whereas SOHLH2 overexpression enhanced this resistance. Immunofluorescence assays further revealed that after X-ray irradiation, γ-H2AX expression was significantly increased in SOHLH2-knockdown A549 cells compared to controls, indicating impaired DNA damage repair capacity; conversely, γ-H2AX expression was significantly decreased in SOHLH2-overexpressing PC9 cells, suggesting enhanced DNA damage repair ability. These findings demonstrate that SOHLH2 promotes radioresistance in NSCLC.

RAD54L, a key factor in homologous recombination (HR), plays an important role in tumor radioresistance [21]. This study explored the interaction between SOHLH2 and RAD54L and their biological functions in NSCLC. Our findings showed that RAD54L overexpression reversed the inhibitory effects of SOHLH2 knockdown on NSCLC cell proliferation and radioresistance. This suggests that SOHLH2 influences NSCLC cell proliferation by regulating RAD54L expression. Moreover, SOHLH2 modulates the activity of the homologous recombination repair pathway via RAD54L regulation, thereby enhancing NSCLC radioresistance. Therefore, the present study investigate the potential function and molecular mechanism of SOHLH2 in NSCLC, particularly in relation to tumor proliferation and radioresistance. These findings may support the development of SOHLH2 as a novel biomarker for radioresistance and as a potential therapeutic target, thereby providing a theoretical basis for future treatment strategies.

Despite elucidating the significant role of SOHLH2 through RAD54L regulation in NSCLC, several limitations remain. Although our results were validated using cell lines and clinical samples, in vivo verification through animal models is lacking. Future studies should include animal experiments to further confirm the functional roles and mechanisms of SOHLH2 regulation of RAD54L in NSCLC proliferation and radioresistance. In summary, this study reveals the high expression of SOHLH2 in NSCLC and its promotion of tumor proliferation and radioresistance, highlighting the molecular mechanism by which SOHLH2 regulates DNA damage repair via RAD54L. These findings not only advance understanding of the fundamental biology of NSCLC but also offer new directions for drug development and therapeutic strategies. Further investigation of the SOHLH2-RAD54L interaction may provide novel targets and approaches for precision treatment of NSCLC.

## Materials and methods

### Tissue samples

A total of 16 pairs of non-small cell lung cancer (NSCLC) tissue samples and corresponding adjacent normal tissues were obtained from archived formalin-fixed paraffin-embedded specimens stored at the First Hospital of Nanchang (The Third Affiliated Hospital of Nanchang University) between 2020 and 2024. All specimens were histopathologically confirmed by the Department of Pathology, based on the WHO (2015) Classification of Tumors of the Lung. This study was approved by the Ethical Review Committee of the First Hospital of Nanchang and informed consents were provided. All procedures involving human participants were conducted in compliance with the Declaration of Helsinki.

### Cell lines

The human NSCLC cell lines A549, H460and PC9, as well as HEK293T cells, all of which exhibit adherent growth characteristics, were used in this study. These cell lines were purchased from the American Type Culture Collection (ATCC, Manassas, VA, USA). Authentication was performed using short tandem repeat (STR) profiling, and all cell lines were confirmed to be free of mycoplasma contamination. Cells were cultured in Dulbecco’s Modified Eagle Medium (DMEM; Thermo Fisher Scientific, Waltham, MA, USA), supplemented with 10% fetal bovine serum (Gibco, Thermo Fisher Scientific). Cultures were maintained in a humidified incubator at 37 °C with 5% CO₂.

### Antibodies

The anti-SOHLH2 antibody (NBP2-20453) was obtained from Novus Biologicals. Anti-Flag (Cat. No. 14793S), phospho-γH2AX (Cat. No. 9718S), and anti-β-actin (Cat. No. 66009-1-Ig) antibodies were purchased from Cell Signaling Technology.

### Bioinformatics analysis

(1) The GEPIA database was used to analyze the mRNA expression of SOHLH2 in NSCLC versus normal lung tissues. (2) The UALCAN database was used to validate differential mRNA expression of SOHLH2 between NSCLC and normal lung tissues.

### Irradiation treatment of cells

Cells were exposed to X-ray irradiation using the Varian Trilogy Linac system (Varian Medical Systems, Palo Alto, CA, USA) at doses ranging from 0 to 6 Gy, with a dose rate of 250 MU/min.

### Quantitative real-time PCR (qRT-PCR)

Total RNA was extracted using an RNA extraction kit (Omega Bio-tek). cDNA synthesis was performed using a reverse transcription kit (Takara), and qRT-PCR was conducted using SYBR Green PCR Master Mix (Takara). Reactions were carried out on a real-time PCR system under the following conditions: 95 °C for 30 s, followed by 40 cycles of 95 °C for 5 s and 60 °C for 34 s; final steps included 60 °C for 1 min and 95 °C for 15 s. Primer sequences were as follows: SOHLH2-Forward: GCTTCCTCAATTATCTGCCAGG；SOHLH2-Reverse: CCCACAGTGACATCTCCAACT；RAD54L-Forward: AGGCAGGTCCTGTGATGATGA；RAD54L-Reverse: TCAAAGGTTTCCGAAAAGGAGAC；GAPDH-Forward: GCACCGTCAAGGCTGAGAAC；GAPDH-Reverse: TGGTGAAGACGCCAGTGGA。GAPDH was used as an internal control, and the relative expression levels of SOHLH2 and RAD54L were calculated using the 2^ − ΔΔCT method.

### Western Blot analysis

Cells were lysed in RIPA buffer (150 mM NaCl, 0.5% EDTA, 50 mM Tris, 0.5% NP-40) on ice for 30 min and centrifuged at 12,000 rpm for 20 min at 4 °C. Protein concentrations were determined using a BCA protein assay kit (Thermo Fisher Scientific, Waltham, MA, USA c). Proteins were separated by 10% SDS-PAGE and transferred to PVDF membranes (Millipore, Billerica, MA, USA). After blocking with 5% non-fat milk for 1 h, membranes were incubated overnight at 4 °C with primary antibodies against SOHLH2 (1:1000), Flag (1:1000), and β-actin (1:1000). HRP-conjugated secondary antibodies were applied at room temperature for 1 h, and bands were visualized using an ECL imaging system Tanon 5200, China.

### Cell proliferation assay

Cells were seeded at 5000 cells per well in 96-well plates and irradiated with 6 Gy X-rays or left untreated. After treatment, 10 µL of CCK-8 reagent was added to each well, followed by a 1-h incubation. Absorbance at 450 nm was measured using a multifunctional microplate reader iMark, Bio-Rad, USA.

### Colony formation assay

Cells (5 × 10³ per well) were seeded into six-well plates and irradiated with the indicated doses. Each group was plated in triplicate. After incubation at 37 °C for 15 days, colonies were washed with PBS, fixed with methanol for 15 min, and stained with 0.1% crystal violet for 60 min. Colonies containing more than 50 cells were counted under a light microscope.

### Immunofluorescence staining

Cells were cultured in glass-bottom dishes (NEST, China), fixed with 4% paraformaldehyde for 15 min at room temperature, and permeabilized with 0.5% Triton X-100. Cells were incubated with phospho-γH2AX primary antibody (1:250, Cell Signaling Technology), followed by a fluorescently labeled secondary antibody. Nuclei were stained with Hoechst 33342 (Thermo Fisher Scientific). Fluorescence images were captured using an Olympus FV12-IXCOV microscope (Olympus, Tokyo, Japan), and double-strand break (DSB) foci were quantified using ImageJ software .

### Dual-luciferase reporter assay

Based on predictions from JASPAR (https://jaspar.elixir.no), potential SOHLH2-binding sites in the 3’-UTR of RAD54L were identified. Wild-type (WT) and mutant (MUT) sequences of RAD54L 3’-UTR were synthesized and cloned into luciferase reporter plasmids. These plasmids (RAD54L-WT, RAD54L-MUT1, and RAD54L-MUT2) were co-transfected with SOHLH2 or vector control into HEK293T cells using Lipofectamine 2000. After 48 h, cells were lysed and luciferase activity was measured. Firefly luciferase activity was normalized to Renilla luciferase activity.

### Homologous recombination (HR) reporter assay

Hela-GFP cells (1 × 10⁶) were electroporated with 12 μg of I-SceI expression plasmid and designated reporter plasmids using the Bio-Rad GenePulser Xcell™ system (Bio-Rad Laboratories, Hercules, CA, USA) at 250 V and 950 μF. GFP expression was assessed 48 h post-transfection by flow cytometry to evaluate HR efficiency.

### Chromatin immunoprecipitation (ChIP) assay

ChIP assays were performed using a commercial kit (Cat. No. 53008, Active Motif, Carlsbad, CA, USA). Cross-linked chromatin was enzymatically digested and sonicated into 200–1000 bp fragments. Immunoprecipitation was conducted using protein G magnetic beads conjugated with anti-SOHLH2 antibody. DNA was purified and analyzed by RT-qPCR. IgG was used as a negative control, and input DNA served as a reference. Primer sequences: ChIP-F: AAGGCAGCTTCTTTCAAGACACC; ChIP-R: GAGTTTGGATTGAATCTACAGTGGTA.

### RNA sequencing (RNA-seq)

RNA-seq was conducted on SOHLH2-knockdown A549 cells by Yuanxin Biopharmaceutical Technology Co., Ltd. (Shanghai, China), including library construction, sequencing, and data analysis. The raw sequence data reported in this paper have been deposited in the Genome Sequence Archive [42] in National Genomics Data Center [43], China National Center for Bioinformation / Beijing Institute of Genomics, Chinese Academy of Sciences (GSA-Human: HRA013776) that are publicly accessible at https://ngdc.cncb.ac.cn/gsa-human.

### Immunohistochemistry (IHC)

Formalin-fixed, paraffin-embedded NSCLC and adjacent normal tissue sections were deparaffinized, rehydrated, and subjected to antigen retrieval and blocking. Primary antibody was incubated overnight at 4 °C, followed by secondary antibody incubation for 30 min and DAB staining. Sections were counterstained, dehydrated, and mounted. IHC images were scanned and analyzed using ImageJ 2.0 (NIH, USA). Evaluation was performed by two experienced pathologists, with discrepancies resolved by consensus.

### Statistical analysis

All experiments were performed in triplicate, and the data are presented as the mean ± SD. Statistical analysis and figure generation were conducted using GraphPad Prism 10. Differences between groups were analyzed using Student’s *t*-test or one-way ANOVA. A *p* < 0.05 was considered statistically significant.

## Supplementary information

Original Data

## Data Availability

Data is provided within the manuscript or supplementary information files.

## References

[1] Li Z, Feiyue Z, Gaofeng L. Traditional Chinese medicine and lung cancer–From theory to practice. Biomed Pharmacother. 2021;137:111381. 33601147 10.1016/j.biopha.2021.111381

[2] Dan A, Burtavel LM, Coman MC, Focsa IO, Duta-Ion S, Juganaru IR, et al. Genetic Blueprints in Lung Cancer: Foundations for Targeted Therapies. Cancers (Basel). 2024;16:4048. 10.3390/cancers16234048 PMC11639944 39682234

[3] Chen W, Zheng R, Baade PD, Zhang S, Zeng H, Bray F, et al. Cancer statistics in China, 2015. CA Cancer J Clin. 2016;66:115–32. 26808342 10.3322/caac.21338

[4] Han B, Zheng R, Zeng H, Wang S, Sun K, Chen R, et al. Cancer incidence and mortality in China, 2022. J Natl Cancer Cent. 2024;4:47–53. 39036382 10.1016/j.jncc.2024.01.006 PMC11256708

[5] Bray F, Laversanne M, Sung H, Ferlay J, Siegel RL, Soerjomataram I, et al. Global cancer statistics 2022: GLOBOCAN estimates of incidence and mortality worldwide for 36 cancers in 185 countries. CA Cancer J Clin. 2024;74:229–63. 38572751 10.3322/caac.21834

[6] Feng H, Xia Y, Wang W, Xu C, Wang Q, Song Z, et al. Expert consensus on the diagnosis and treatment of non-small cell lung cancer with MET alteration. Cancer Biol Med. 2025;22:237–65. 40200811 10.20892/j.issn.2095-3941.2024.0503 PMC11976709

[7] Zappa C, Mousa SA. Non-small cell lung cancer: current treatment and future advances. Transl Lung Cancer Res. 2016;5:288–300. 27413711 10.21037/tlcr.2016.06.07 PMC4931124

[8] Chen P, Liu Y, Wen Y, Zhou C. Non-small cell lung cancer in China. Cancer Commun (Lond). 2022;42:937–70. 36075878 10.1002/cac2.12359 PMC9558689

[9] Riely GJ, Wood DE, Ettinger DS, Aisner DL, Akerley W, Bauman JR, et al. Non-Small Cell Lung Cancer, Version 4.2024, NCCN Clinical Practice Guidelines in Oncology. J Natl Compr Canc Netw. 2024;22:249–74. 38754467 10.6004/jnccn.2204.0023

[10] Cheng M, Jolly S, Quarshie WO, Kapadia N, Vigneau FD, Kong FS. Modern Radiation Further Improves Survival in Non-Small Cell Lung Cancer: An Analysis of 288,670 Patients. J Cancer. 2019;10:168–77. 30662537 10.7150/jca.26600 PMC6329848

[11] Tang L, Wei F, Wu Y, He Y, Shi L, Xiong F, et al. Role of metabolism in cancer cell radioresistance and radiosensitization methods. J Exp Clin Cancer Res. 2018;37:87. 29688867 10.1186/s13046-018-0758-7 PMC5914062

[12] Suzuki H, Ahn HW, Chu T, Bowden W, Gassei K, Orwig K, et al. SOHLH1 and SOHLH2 coordinate spermatogonial differentiation. Dev Biol. 2012;361:301–12. 22056784 10.1016/j.ydbio.2011.10.027 PMC3249242

[13] Hao J, Yamamoto M, Richardson TE, Chapman KM, Denard BS, Hammer RE, et al. Sohlh2 knockout mice are male-sterile because of degeneration of differentiating type A spermatogonia. Stem Cells. 2008;26:1587–97. 18339773 10.1634/stemcells.2007-0502

[14] Zhang T, Murphy MW, Gearhart MD, Bardwell VJ, Zarkower D. The mammalian Doublesex homolog DMRT6 coordinates the transition between mitotic and meiotic developmental programs during spermatogenesis. Development. 2014;141:3662–71. 25249458 10.1242/dev.113936 PMC4197572

[15] Ballow DJ, Xin Y, Choi Y, Pangas SA, Rajkovic A. Sohlh2 is a germ cell-specific bHLH transcription factor. Gene Expr Patterns. 2006;6:1014–8. 16765102 10.1016/j.modgep.2006.04.007

[16] Ji S, Zhang W, Zhang X, Hao C, Hao A, Gao Q, et al. Sohlh2 suppresses epithelial to mesenchymal transition in breast cancer via downregulation of IL-8. Oncotarget. 2016;7:49411–24. 27384482 10.18632/oncotarget.10355 PMC5226517

[17] Cui W, Xiao Y, Zhang R, Zhao N, Zhang X, Wang F, et al. SOHLH2 Suppresses Angiogenesis by Downregulating HIF1alpha Expression in Breast Cancer. Mol Cancer Res. 2021;19:1498–509. 34158392 10.1158/1541-7786.MCR-20-0771

[18] Uhrig ME, Sharma N, Maxwell P, Gomez J, Selemenakis P, Mazin AV, et al. Disparate requirements for RAD54L in replication fork reversal. Nucleic Acids Res. 2024;52:12390–404. 39315725 10.1093/nar/gkae828 PMC11551752

[19] Ceballos SJ, Heyer WD. Functions of the Snf2/Swi2 family Rad54 motor protein in homologous recombination. Biochim Biophys Acta. 2011;1809:509–23. 21704205 10.1016/j.bbagrm.2011.06.006 PMC3171615

[20] Bhoir S, Ogundepo O, Yu X, Shi R, De Benedetti A. Exploiting TLK1 and Cisplatin Synergy for Synthetic Lethality in Androgen-Insensitive Prostate Cancer. Biomedicines. 2023;11:2987. 10.3390/biomedicines11112987 PMC10669050 38001987

[21] Li Q, Xie W, Wang N, Li C, Wang M. CDC7-dependent transcriptional regulation of RAD54L is essential for tumorigenicity and radio-resistance of glioblastoma. Transl Oncol. 2018;11:300–6. 29413763 10.1016/j.tranon.2018.01.003 PMC5884092

[22] Cheng C, Pei X, Li SW, Yang J, Li C, Tang J, et al. CRISPR/Cas9 library screening uncovered methylated PKP2 as a critical driver of lung cancer radioresistance by stabilizing beta-catenin. Oncogene. 2021;40:2842–57. 33742119 10.1038/s41388-021-01692-x

[23] Mun JY, Baek SW, Park WY, Kim WT, Kim SK, Roh YG, et al. E2F1 Promotes Progression of Bladder Cancer by Modulating RAD54L Involved in Homologous Recombination Repair. Int J Mol Sci. 2020;21:9025. 10.3390/ijms21239025 PMC7730422 33261027

[24] Bai X, Wang J, Huo L, Xie Y, Xie W, Xu G, et al. Serine/Threonine Kinase CHEK1-Dependent Transcriptional Regulation of RAD54L Promotes Proliferation and Radio Resistance in Glioblastoma. Transl Oncol. 2018;11:140–6. 29287241 10.1016/j.tranon.2017.11.007 PMC6002345

[25] Zhang S, Chen X, Jin E, Wang A, Chen T, Zhang X, et al. The GSA Family in 2025: A Broadened Sharing Platform for Multi-omics and Multimodal Data. Genomics Proteomics Bioinformatics. 2025;23:qzaf072. 10.1093/gpbjnl/qzaf072 PMC12451262 40857552

[26] Members C-N. Partners. Database Resources of the National Genomics Data Center, China National Center for Bioinformation in 2025. Nucleic Acids Res. 2025;53:D30–D44. 39530327 10.1093/nar/gkae978 PMC11701749

[27] Sutherland BM, Bennett PV, Sidorkina O, Laval J, Clustered DNA. damages induced in isolated DNA and in human cells by low doses of ionizing radiation. Proc Natl Acad Sci USA. 2000;97:103–8. 10618378 10.1073/pnas.97.1.103 PMC26623

[28] Li L, Zhu T, Gao YF, Zheng W, Wang CJ, Xiao L, et al. Targeting DNA Damage Response in the Radio(Chemo)therapy of Non-Small Cell Lung Cancer. Int J Mol Sci. 2016;17:839. 10.3390/ijms17060839 PMC4926373 27258253

[29] Pouliliou S, Koukourakis MI. Gamma histone 2AX (gamma-H2AX)as a predictive tool in radiation oncology. Biomarkers. 2014;19:167–80. 24611829 10.3109/1354750X.2014.898099

[30] Sharma A, Singh K, Almasan A. Histone H2AX phosphorylation: a marker for DNA damage. Methods Mol Biol. 2012;920:613–26. 22941631 10.1007/978-1-61779-998-3_40

[31] Cai S, Yang W, Xing H, Yang J, Luo H, Ye X. Bibliometric analysis of current trends and emerging patterns in the application of nanomaterials for non-small cell lung cancer. Discov Oncol. 2025;16:802. 40382731 10.1007/s12672-025-02602-3 PMC12086128

[32] Liu C, Wang S, Zheng S, Xu F, Cao Z, Feng X, et al. Avoiding Absolute Quantification Trap: A Novel Predictive Signature of Clinical Benefit to Anti-PD-1 Immunotherapy in Non-Small Cell Lung Cancer. Front Immunol. 2021;12:782106. 34868057 10.3389/fimmu.2021.782106 PMC8640493

[33] Tian S. Identification of monotonically differentially expressed genes for non-small cell lung cancer. BMC Bioinformatics. 2019;20:177. 30971213 10.1186/s12859-019-2775-8 PMC6458730

[34] Chen X, Zeng C. Pioneering the Way: The Revolutionary Potential of Antibody-Drug Conjugates in NSCLC. Curr Treat Options Oncol. 2024;25:556–84. 38520605 10.1007/s11864-024-01196-2

[35] Yoon SM, Shaikh T, Hallman M. Therapeutic management options for stage III non-small cell lung cancer. World J Clin Oncol. 2017;8:1–20. 28246582 10.5306/wjco.v8.i1.1 PMC5309711

[36] Yun M, Yingzi L, Jie G, Guanxin L, Zimei Z, Zhen C, et al. PPDPF Promotes the Progression and acts as an Antiapoptotic Protein in Non-Small Cell Lung Cancer. Int J Biol Sci. 2022;18:214–28. 34975328 10.7150/ijbs.65654 PMC8692159

[37] Zhang X, Liu X, Cui W, Zhang R, Liu Y, Li Y, et al. Sohlh2 alleviates malignancy of EOC cells under hypoxia via inhibiting the HIF1alpha/CA9 signaling pathway. Biol Chem. 2020;401:263–71. 31318683 10.1515/hsz-2019-0119

[38] Zhang H, Hao C, Wang Y, Ji S, Zhang X, Zhang W, et al. Sohlh2 inhibits human ovarian cancer cell invasion and metastasis by transcriptional inactivation of MMP9. Mol Carcinog. 2016;55:1127–37. 26153894 10.1002/mc.22355

[39] Zhang H, Zhang X, Ji S, Hao C, Mu Y, Sun J, et al. Sohlh2 inhibits ovarian cancer cell proliferation by upregulation of p21 and downregulation of cyclin D1. Carcinogenesis. 2014;35:1863–71. 24858206 10.1093/carcin/bgu113

[40] Liu Y, Cui W, Zhang R, Zhi S, Liu L, Liu X, et al. Sohlh2 Inhibits the Malignant Progression of Renal Cell Carcinoma by Upregulating Klotho via DNMT3a. Front Oncol. 2021;11:769493. 35127476 10.3389/fonc.2021.769493 PMC8807643

[41] Yang Z, Wu H, Zhang K, Rao S, Qi S, Liu M, et al. Circ_0007580 knockdown strengthens the radiosensitivity of non-small cell lung cancer via the miR-598-dependent regulation of THBS2. Thorac Cancer. 2022;13:678–89. 35044104 10.1111/1759-7714.14221 PMC8888153

[42] Huang Q. Predictive relevance of ncRNAs in non-small-cell lung cancer patients with radiotherapy: a review of the published data. Biomark Med. 2018;12:1149–59. 30191721 10.2217/bmm-2018-0004

[43] Zhou T, Zhang LY, He JZ, Miao ZM, Li YY, Zhang YM, et al. Review: Mechanisms and perspective treatment of radioresistance in non-small cell lung cancer. Front Immunol. 2023;14:1133899. 36865554 10.3389/fimmu.2023.1133899 PMC9971010

