# Supplementary material for: SOHLH2-RAD54L axis induces radioresistance by promoting homologous recombination repair in non-small cell lung cancer
Source: Cell Death Discov. 2026 Jan 14;12:84. doi: 10.1038/s41420-025-02924-9 (PMC12876998; doi:10.1038/s41420-025-02924-9)

Full-length Western blot images corresponding to Figures 2A in the main manuscript.

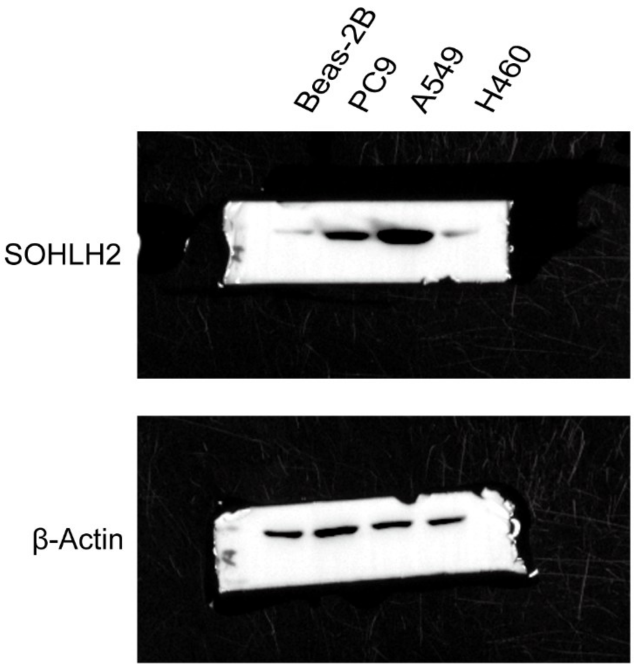

Full-length Western blot images corresponding to Figures 2C in the main manuscript.

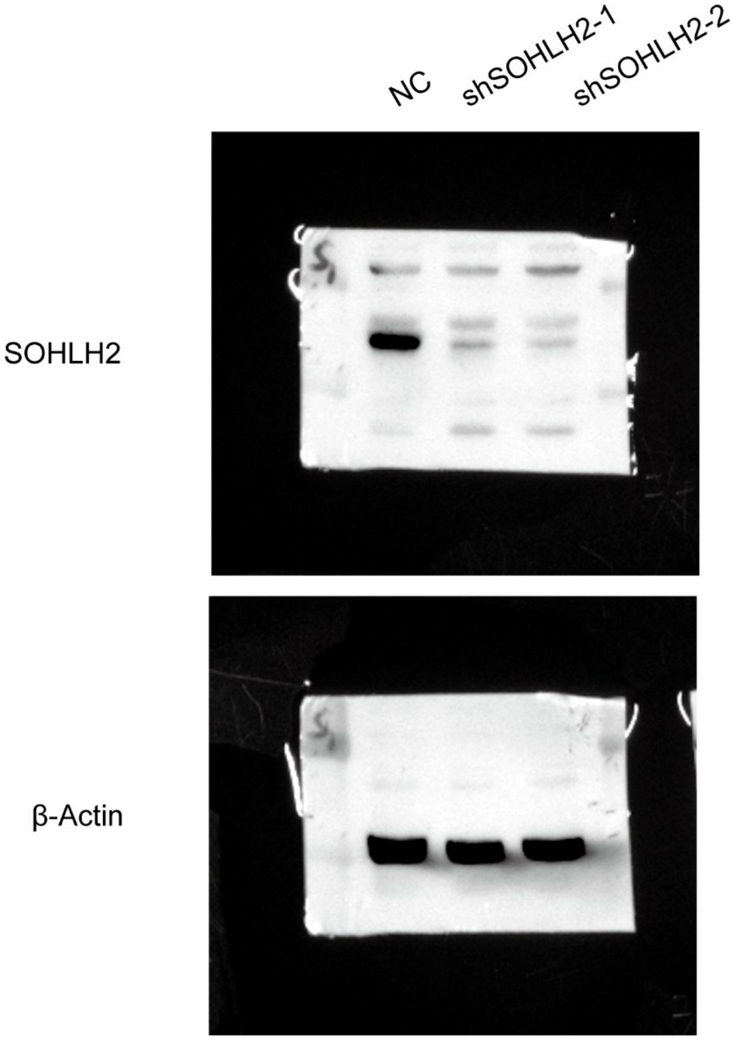

Full-length Western blot images corresponding to Figures 2E in the main manuscript.

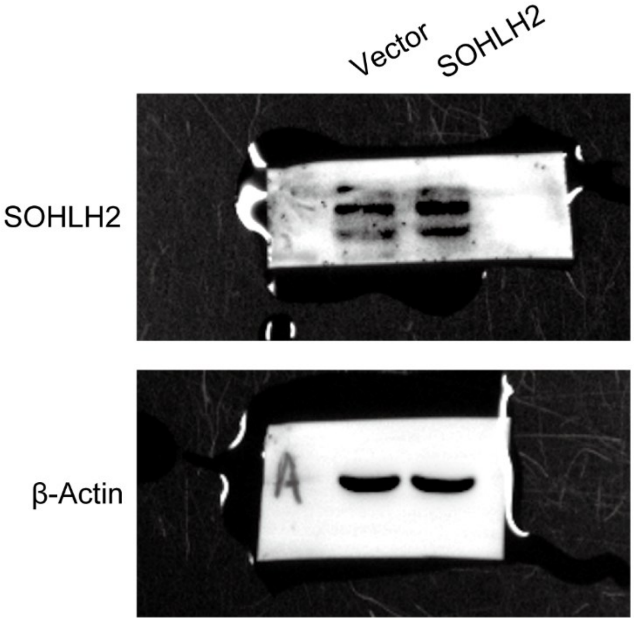

Full-length Western blot images corresponding to Figures 2G in the main manuscript.

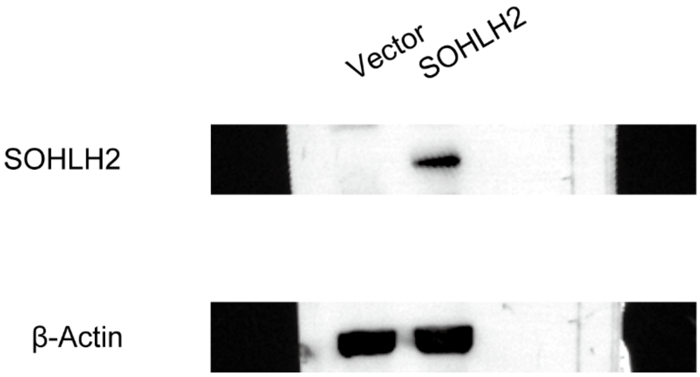

Full-length Western blot images corresponding to Figures 5B in the main manuscript.

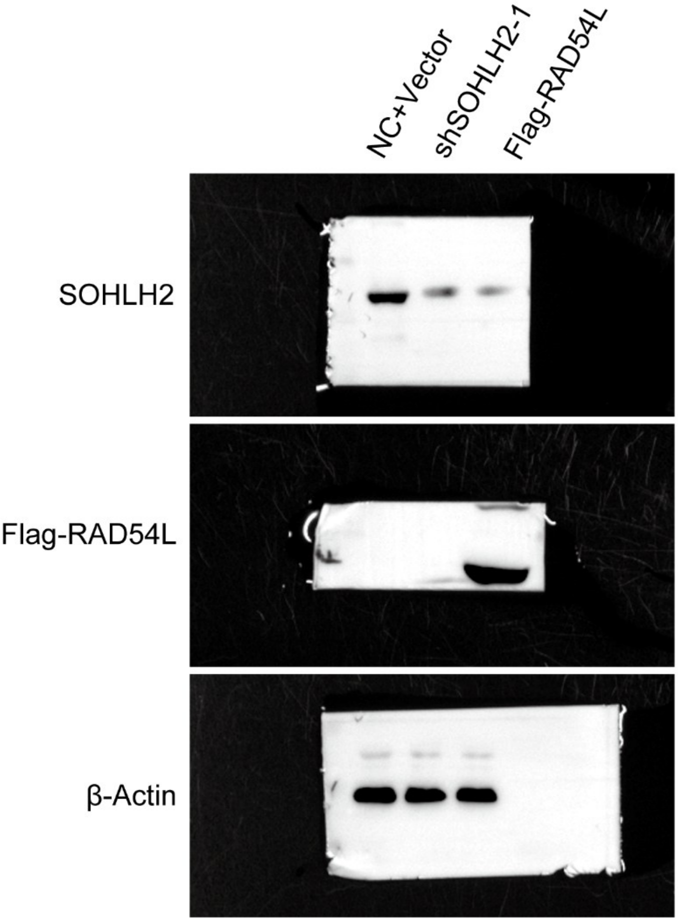

Supplement: Supplementary file 1 — Original Data [file 41420_2025_2924_MOESM1_ESM.pdf]
